# Temporal evolution of non-contrast CT markers of expansion relates to the dynamics of acute intracerebral hemorrhage

**DOI:** 10.1007/s00234-025-03789-6

**Published:** 2025-10-17

**Authors:** David Rodriguez-Luna, Olalla Pancorbo, João André Sousa, Renato Simonetti, Pilar Coscojuela, Marc Rodrigo-Gisbert, Federica Rizzo, Marta Olivé-Gadea, Manuel Requena, Álvaro García-Tornel, Noelia Rodriguez-Villatoro, Jesús M. Juega, Marián Muchada, Jorge Pagola, Marta Rubiera, Marc Ribo, Alejandro Tomasello, Carlos A. Molina

**Affiliations:** 1 Stroke Research Group, Vall d’Hebron Research Institute, Barcelona, Spain; 2 Stroke Unit, Department of Neurology, Vall d’Hebron University Hospital, Barcelona, Spain; 3 Department of Neuroradiology, Vall d’Hebron University Hospital, Barcelona, Spain

**Keywords:** Humans, Stroke, Cerebral hemorrhage, Tomography, x-ray computed, Computed tomography angiography

## Abstract

**Purpose:**

The temporal evolution of non-contrast CT (NCCT) markers of intracerebral hemorrhage (ICH) expansion during the dynamics of acute ICH is understudied. We aimed to evaluate the temporal evolution of these markers and its relationship with ICH dynamics.

**Methods:**

Single-center, prospective, observational cohort study on 271 ICH patients < 6 h. Patients underwent baseline NCCT and multiphase CTA, and 24-hour NCCT. NCCT markers included: irregular shape, satellite sign, and island sign (shape markers); heterogeneous density, hypodensities, swirl sign, black hole sign, blend sign, and fluid level (qualitative density markers); and mean, standard deviation, and coefficient of variation hematoma density (quantitative density markers). The spot sign in first phase of multiphase CTA was considered marker of active hemorrhage. Primary outcome was the change in frequency or values of NCCT markers from baseline to follow-up NCCT. Other outcomes included associations of active hemorrhage with NCCT markers at baseline and with the magnitude of their change at follow-up NCCT.

**Results:**

Heterogeneous density predicted active hemorrhage with the highest accuracy (66.4%); hypodensities had the highest AUC (0.626, 95% CI 0.561–0.691). From baseline to follow-up NCCT, the frequency of heterogeneous density (54 [27.8%] vs. 24 [12.4%], *p* < 0.001) and hypodensities (82 [42.3%] vs. 52 [26.8%], *p* < 0.001) decreased, with greater reductions when active hemorrhage at baseline (17 [29.0%] vs. 12 [10.0%], *p* = 0.001; and 15 [26.3%] vs. 13 [10.8%], *p* = 0.008; respectively).

**Conclusion:**

Heterogeneous density and hypodensities are the markers most closely related to acute ICH dynamics, better predicting active hemorrhage at baseline and decreasing with hematoma stabilization.

**Supplementary Information:**

The online version contains supplementary material available at 10.1007/s00234-025-03789-6.

## Introduction

Hematoma expansion is a potentially modifiable determinant of poor outcome that represents an appealing therapeutic target in patients with acute intracerebral hemorrhage (ICH) [1]. Accurately identifying individuals at high risk of hematoma expansion is crucial for the success of preventive strategies. While the single-phase CTA spot sign is a robust predictor of hematoma expansion [2], multiphase CTA offers improved risk stratification [3]. Furthermore, the presence of a spot sign in earlier CTA acquisitions is considered indicative of active hemorrhage [3, 4]. However, there is growing interest in exploring non-contrast CT (NCCT) markers of expansion because NCCT is more readily available compared to CTA [5–7].

Various NCCT markers have been proposed as potential predictors of hematoma expansion, encompassing qualitative assessments of hematoma shape and density alongside quantitative evaluations [8, 9]. Although the presence of these markers on baseline NCCT has been linked to an increased risk of expansion, and it is thought to be that hematoma irregularity and heterogeneity indicate an early-intermediate stage of hematoma maturity with active hemorrhage, the temporal evolution of these markers during the dynamic process of acute ICH remains understudied [8].

We hypothesized that NCCT markers of hematoma expansion evident at baseline NCCT are indicative to active hemorrhage, and thus, their presence would change on follow-up NCCT after ICH stabilization. The main objective of this study was to evaluate the temporal evolution of different NCCT markers of hematoma expansion in acute ICH patients. Additionally, we aimed to investigate the significance of these markers by determining their association with active hemorrhage at baseline and exploring whether changes in NCCT markers over time are associated with the time from symptom onset and active hemorrhage.

## Materials and methods

### Study design

**Fig. 1:**
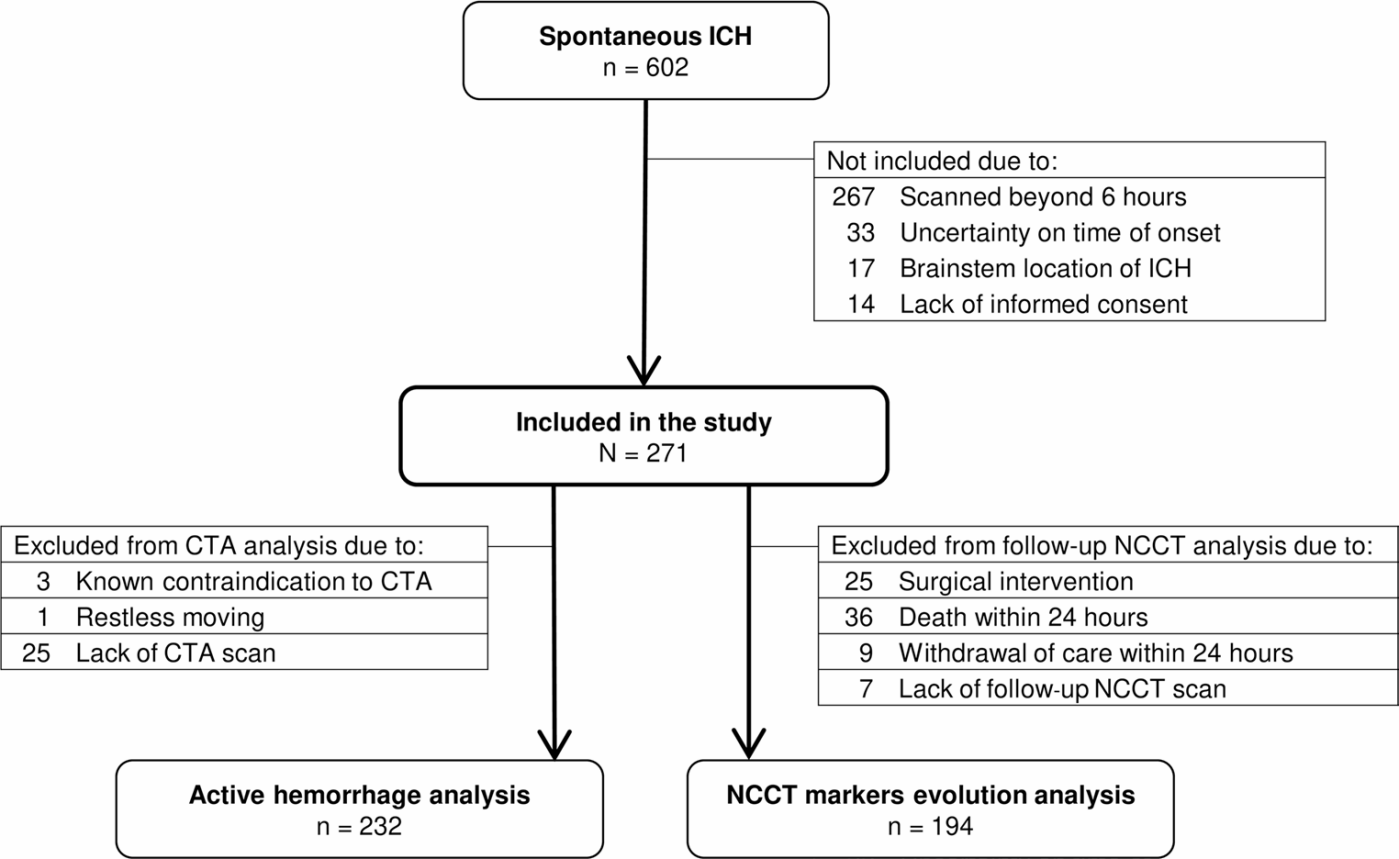
Cohort flowchart. CTA, computed tomography angiography; ICH, intracerebral hemorrhage; NCCT, non-contrast computed tomography

We conducted a single-center, prospective, observational cohort study of consecutive subjects aged ≥ 18 years with spontaneous ICH without a visualized structural cause scanned within 6 h from symptom onset during a 4.5-year period (January 1, 2018, to June 30, 2022). Exclusion criteria included uncertainty about the exact time from symptom onset and brainstem ICH location (Fig. 1).

### Ethics approval and patient consents

The local ethics committee approved all aspects of the study protocol. Participants or their next of kin provided informed consent. The report of this study follows the Strengthening the Reporting of Observational Studies in Epidemiology (STROBE) reporting guidelines (Supplementary Material 1) [10].

### Data acquisition

On admission, we recorded relevant demographic characteristics, medical history, clinical presentation, neurological status, and routine laboratory tests. Participants underwent a standard institutional acute ICH CT protocol, which included a baseline NCCT followed by a multiphase CTA (< 6 h from symptom onset), and a follow-up NCCT at 24 (−2/+6) hours [3]. All scans were performed using a multidetector CT scanner (SOMATOM Definition AS; Siemens). Images were acquired with 1.0-mm section thickness in NCCT scans and with 0.6-mm section thickness in multiphase CTA scans. Multiphase CTA was performed in three automated phases after intravenous contrast injection: the first phase was acquired 8 s after the CT attenuation value of the descending aorta reached the threshold value of 120 Hounsfield units; the second phase after a delay of 4 s, and the third after a delay of 15 s [3].

### Image analysis

An experienced stroke neurologist (DRL) blinded to multiphase CTA scans and spot sign status prospectively evaluated the NCCT scans. Baseline and follow-up NCCT scans were evaluated in two separate reading sessions, with a minimum of 14 days between them to eliminate recall bias. ICH volumes were measured using semiautomatic Hounsfield-unit, threshold-based, computerized planimetry software (Syngo; Siemens). The onset-to imaging time (OIT), defined as the time from symptom onset to the baseline NCCT scan, and the location of the ICH were recorded.

**Fig. 2:**
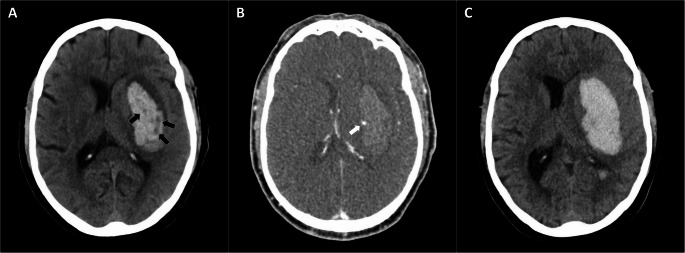
Illustrative example of evolution of non-contrast CT markers of hematoma expansion and its relationship with active hemorrhage. **(A)** Patient with intracerebral hemorrhage (ICH) volume of 26.6 mL on baseline non-contrast CT (taken at 71 min from symptom onset) showing heterogeneous density and hypodensities (black arrows). **(B)** spot sign observed in phase 1 of multiphase CT angiography as marker of active hemorrhage (white arrow). **(C)** Hematoma expansion on follow-up non-contrast CT (ICH volume of 48.8 mL) without the presence of either heterogeneous density or hypodensities

NCCT markers of hematoma expansion were evaluated on both baseline and follow-up NCCT scans (Fig. 2): irregular shape, satellite sign, and island sign were evaluated as shape markers [11–13]; heterogeneous density, hypodensities, swirl sign, black hole sign, blend sign, and fluid level as qualitative density markers [11, 14–18]; and mean, standard deviation (SD), and coefficient of variation (CV) hematoma density as quantitative density markers [9, 19]. Shape and qualitative density markers were defined according to previously standardized diagnostic criteria [8]. Mean and SD of mean hematoma density were automatically calculated from the Hounsfield unit attenuation of ICH using the semiautomatic computerized planimetry software used in ICH volumetric analysis (Syngo; Siemens). CV hematoma density was calculated as SD/mean×100 [19].

An experienced neuroradiologist (PC) blinded to follow-up NCCT scans and clinical outcomes independently interpreted the multiphase CTA scans. The presence of spot signs was recorded in phases 1, 2, and 3 of multiphase CTA. The spot sign was defined according to previously established criteria [20]. The presence of the spot sign in phase 1 of multiphase CTA was evaluated as marker of active hemorrhage [3].

To assess the interrater reliability of NCCT and multiphase CTA analyses, a stroke fellow (JAS) independently assessed NCCT markers of hematoma expansion, ICH volumes, and multiphase CTA spot sign status in 30 randomly selected scans.

### Outcomes

The primary outcome was the temporal evolution of NCCT markers of hematoma expansion from baseline to follow-up NCCT scans. This evolution was assessed by changes in the frequency of shape and qualitative density markers, as well as changes in the values of quantitative density markers.

Secondary outcomes included the associations of the magnitude of change of NCCT markers with active hemorrhage and OIT. The magnitude of change of NCCT markers was defined as the difference (delta) in frequency of shape and qualitative density markers, or in the value of quantitative density markers. Additionally, secondary outcomes involved examining associations of NCCT markers at baseline with OIT, ICH volume, active hemorrhage, and hematoma expansion. Hematoma expansion was defined at follow-up NCCT as an ICH absolute growth > 6 mL or a relative enlargement > 33% from baseline NCCT [7, 21].

### Statistical analysis

The statistical analysis was performed using R 3.6.2 statistical Software (R Foundation for Statistical Computing). The continuous variables were assessed for normality using the Shapiro-Wilk test and are presented as medians (interquartile intervals). Estimates of interrater reliability for NCCT markers and multiphase CTA spot sign status were calculated using the unweighted *κ* statistic, while for ICH volumetric analyses, it was calculated using a two-way random-effects ANOVA, and expressed as intraclass correlation coefficient (ICC) [22]. Statistical significance for intergroup differences was assessed by Pearson *χ*^2^ or Fisher’s exact tests for categorical variables and by Mann-Whitney *U* test for continuous variables. Correlations were assessed using Spearman’s correlation coefficient. The sensitivity, specificity, predictive values, accuracy, and area under the receiver operating characteristic curve (AUROC) of each marker in the prediction of active hemorrhage were calculated. For this purpose, quantitative density markers were dichotomized using their median values. The DeLong test was used to test for differences in AUROC values. Significant differences between markers from baseline to follow-up NCCT scans were assessed using McNemar and exact McNemar tests for shape and qualitative density markers, and the Wilcoxon signed-rank test for quantitative density markers. A two-sided *p*-value of < 0.05 was considered significant for all tests.

## Results

### Study population

A total of 271 participants (71.3 ± 14.0 years, 166 [61.3%] male) were included in the study (Fig. 1). Baseline characteristics and the frequency of the different NCCT markers at baseline are summarized in Table 1. The interrater reliability was moderate-to-excellent for NCCT markers of hematoma expansion and multiphase CTA analyses (ranging from ICC 0.651, 95% CI 0.021–1.000, to ICC 0.933, 95% CI 0.805–1.000), and excellent for ICH volumetric analysis (ICC 0.992; 95% CI 0.983–0.996) in the 30 randomly selected NCCT and multiphase CTA scans.

**Table 1:** Baseline characteristics and frequency of NCCT markers and multiphase CTA spot signs, and ICH volume and hematoma expansion on follow-up NCCT

		All Patients (*N* = 271)
Baseline characteristics		
Age (y)		71.3 ± 14.0
Male sex		166 (61.3)
Antiplatelet drugs use		64 (23.6)
Anticoagulant drugs use		48 (17.7)
GCS score		15 (11–15)
NIHSS score		14 (8–20)
Onset to imaging time (min)		120 (78–214)
ICH volume (mL)		15.4 (6.2–38.3)
Lobar ICH location		94 (34.7)
Intraventricular extension		98 (36.2))
Subarachnoid extension		68 (25.1)
Baseline shape markers		
Irregular shape		188 (69.4)
Satellite sign		59 (21.8)
Island sign		136 (50.2)
Baseline qualitative density markers		
Heterogeneous density		83 (30.6)
Hypodensities		129 (47.6)
Swirl sign		131 (48.3)
Black hole sign		14 (5.2)
Blend sign		28 (10.3)
Fluid level		8 (3.0)
Baseline quantitative density markers		
Mean ICH density (HU)		63.6 (60.3–66.6)
SD ICH density (HU)		9.2 (8.4–10.0)
CV ICH density		14.3 (13.2–15.6)
Baseline multiphase CTA spot signs		
Spot sign in phase 1 ^a^		84 (36.2)
Spot sign in phase 2 ^b^		95 (44.2)
Spot sign in phase 3 ^b^		100 (46.5)
Follow-up ICH volume ^c^		
ICH volume (mL)		12.5 (5.3–31.3)
Absolute hematoma expansion (mL)		0.6 (0.0–4.0)
Relative hematoma expansion (%)		8.6 (−0.4–25.4)

Values are presented as n (%), mean ± SD, or median (IQR)

*CV* coefficient of variation, *GCS* Glasgow Coma Scale, *HU* Hounsfield units, *ICH* intracerebral hemorrhage, *NIHSS* National Institutes of Health Stroke Scale, *SD* standard deviation

^a^*n*=232

^b^*n*=215

^c^*n*=194

### ICH volume, onset-to-imaging time, multiphase CTA spot signs, and presence of active hemorrhage

The median ICH volume was 15.4 (6.2–38.3) mL, and the median OIT 120 (78–213) minutes. With the exception of fluid level and SD hematoma density, all other markers were significantly related to baseline ICH volume (*p* < 0.005 for all comparisons). A lower OIT was related to irregular shape (111 [77–189] vs. 145 [86–245] minutes, *p* = 0.020), as well as correlated to lower mean (*ρ* = 0.248, *p* = 0.005) and SD (*ρ* = 0.159, *p* = 0.010) hematoma density. Conversely, satellite, island sign, all qualitative density markers, and CV hematoma density were not related to OIT (*p* > 0.005 for all comparisons).

The spot sign in phase 1 of multiphase CTA was present in 84 (36.2%) out of the 232 participants included in active hemorrhage analysis (Fig. 1). Heterogeneous density and lower CV hematoma density were exclusively associated with the presence of spot sign in phase 1 of multiphase CTA, while not in the others (Table S1). Conversely, all shape markers, hypodensities, swirl sign, and lower SD hematoma density were related to the presence of spot signs across all phases of multiphase CTA (Table S1).

The diagnostic performance of the different markers in predicting active hemorrhage at baseline is shown in Table 2. Heterogeneous density was the marker that predicted the presence of active hemorrhage with the highest accuracy (66.4%), while hypodensities showed the highest AUROC (0.626, 95% CI 0.561–0.691). Both heterogeneous density and hypodensities had a significantly higher AUROC in the prediction of active hemorrhage than black hole sign (*p* = 0.008 and *p* = 0.005), blend sign (*p* = 0.025 and *p* = 0.017), and fluid level (*p* < 0.001 and *p* < 0.001), respectively. Conversely, the AUROC was not significantly different between heterogeneous density and hypodensities, nor between other markers comparisons (*p* > 0.050 for all comparisons).

**Table 2:** Diagnostic performance of NCCT markers in the prediction of the presence of active hemorrhage (*n* = 232)

		Sensitivity, %	Specificity, %	PPV, %	NPV, %	Accuracy, %	AUROC (95% CI)
Shape markers							
Irregular shape		82.1 (69/84)	39.9 (59/148)	43.7 (69/158)	79.7 (59/74)	55.2 (128/232)	0.610 (0.553–0.667)
Satellite sign		33.3 (28/84)	83.1 (123/148)	52.8 (28/53)	68.7 (123/179)	65.1 (151/232)	0.582 (0.523–0.641)
Island sign		65.5 (55/84)	59.5 (88/148)	47.8 (55/115)	75.2 (88/117)	61.6 (143/232)	0.625 (0.560–0.689)
Qualitative density markers							
Heterogeneous density		44.0 (37/84)	79.1 (117/148)	54.4 (37/68)	71.3 (117/164)	66.4 (154/232)	0.616 (0.553–0.678)
Hypodensities		63.1 (53/84)	62.2 (92/148)	48.6 (53/109)	74.8 (92/123)	62.5 (145/232)	0.626 (0.561–0.691)
Swirl sign		61.9 (52/84)	59.5 (88/148)	46.4 (52/112)	73.3 (88/120)	60.3 (140/232)	0.607 (0.541–0.672)
Black hole sign		8.3 (7/84)	97.3 (144/148)	63.6 (7/11)	65.2 (144/221)	65.1 (151/232)	0.528 (0.496–0.560)
Blend sign		14.3 (12/84)	91.2 (135/148)	48.0 (12/25)	65.2 (135/207)	63.4 (147/232)	0.528 (0.484–0.572)
Fluid level		2.4 (2/84)	97.3 (144/148)	33.3 (2/6)	63.7 (144/226)	62.9 (146/232)	0.498 (0.477–0.519)
Quantitative density markers							
Mean ICH density < 63.5 HU^a^		54.8 (46/84)	52.7 (78/148)	39.7 (46/116)	67.2 (78/116)	53.4 (124/232)	0.537 (0.470–0.604)
SD ICH density < 9.1 HU^a^		63.1 (53/84)	55.8 (82/147)	44.9 (53/118)	72.6 (82/113)	58.4 (135/231)	0.594 (0.529–0.660)
CV ICH density < 14.4^a^		61.9 (52/84)	56.5 (83/147)	44.8 (52/116)	72.2 (83/115)	58.4 (135/231)	0.592 (0.526–0.658)

Numbers in parentheses are raw data unless otherwise indicated

*AUROC* area under the receiver-operating characteristic curve, *ICH* intracerebral hemorrhage, *NCCT* non-contrast computed tomography, *NPV *negative predictive value, *PPV *positive predictive value

^*a*^Values below the median value of quantitative density markers

### Evolution of NCCT markers and hematoma expansion

The evolution of NCCT markers in the 194 participants included in NCCT markers evolution analysis (Fig. 1) is provided in Table 3. Compared with baseline NCCT, the frequency of heterogeneous density, hypodensities, swirl sign, and blend sign was lower at the follow-up NCCT, while both mean and SD hematoma density were higher. Contrarily, the frequency of irregular shape was higher at the follow-up NCCT. Hematoma expansion occurred in 49 (25.3%) participants. Irregular shape, heterogeneous density, hypodensities, and blend sign were significantly associated with hematoma expansion, while all other NCCT markers were not (Table 3).

**Table 3:** Evolution of NCCT markers from baseline to follow-up NCCT scan and association of NCCT markers at baseline with hematoma expansion (*n* = 194)

		Presence of NCCT Markers			Hematoma Expansion		
		Baseline NCCT	Follow-up NCCT	*p* Value^a^	Yes (*n* = 49)	No (*n* = 145)	*p* Value^a^
Shape markers							
Irregular shape		121 (62.4)	128 (66.0)	0.046	39 (79.6)	82 (56.6)	0.004
Satellite sign		37 (19.1)	37 (19.1)	0.999	12 (24.5)	25 (17.2)	0.264
Island sign		81 (41.8)	83 (42.8)	0.724	23 (46.9)	58 (40.0)	0.394
Qualitative density markers							
Heterogeneous density		54 (27.8)	24 (12.4)	< 0.001	20 (40.8)	34 (23.4)	0.019
Hypodensities		82 (42.3)	52 (26.8)	< 0.001	27 (55.1)	55 (37.9)	0.035
Swirl sign		91 (46.9)	52 (26.8)	< 0.001	26 (53.1)	65 (44.8)	0.318
Black hole sign		8 (4.1)	2 (1.0)	0.070	2 (4.1)	6 (4.1)	0.999
Blend sign		20 (10.3)	8 (4.1)	0.002	11 (22.4)	9 (6.2)	0.005
Fluid level		4 (2.1)	9 (4.6)	0.063	1 (2.0)	3 (2.1)	0.999
Quantitative density markers							
Mean ICH density (HU)		62.6 (59.9–66.0)	64.6 (62.2–67.5)	< 0.001	61.9 (60.0–65.9.0.9)	62.7 (59.9–66.1)	0.585
SD ICH density (HU)		9.2 (8.4–10.0)	9.6 (8.8–10.4)	< 0.001	8.9 (8.1–10.0)	9.2 (8.5–10.0)	0.366
CV ICH density		14.6 (13.4–15.8)	14.9 (13.8–16.0)	0.139	14.6 (13.5–16.1)	14.6 (13.4–15.8)	0.768

Values are presented as n (%) or median (IQR)

*CV* coefficient of variation, *HU* Hounsfield units, *ICH* intracerebral hemorrhage, *NCCT* non-contrast computed tomography, *SD* standard deviation

^a^*p* values are from McNemar, McNemar exact, Wilcoxon signed-rank, Pearson *χ*^2^, Fisher’s exact, and Mann-Whitney *U* tests

The magnitude of change in quantitative density markers from baseline to follow-up NCCT was inversely correlated with OIT, including mean hematoma density (*ρ*=−0.241, *p* = 0.001), SD hematoma density (*ρ*=−0.245, *p* = 0.001), and CV hematoma density (*ρ*=−0.191, *p* = 0.008), yet the change in the frequency of shape and qualitative density markers was not significantly related to OIT (*p* > 0.050 for all comparisons). The presence of active hemorrhage at baseline associated a higher decrease in the frequency of heterogeneous density, hypodensities, swirl sign, and blend sign from baseline to follow-up NCCT, as well as a higher increase in mean, SD, and CV hematoma density (Table 4).

**Table 4:** Association of the magnitude of the change in markers from baseline to follow-up NCCT with the presence of active hemorrhage at baseline in patients who underwent both multiphase CTA and follow-up NCCT scans

			Active Hemorrhage		
		All patients (*n* = 177)	Yes (*n* = 57)	No (*n* = 120)	*p* Value^a^
Shape markers					
Irregular shape		9 (5.1)	5 (8.8)	4 (3.3)	0.150
Satellite sign		8 (4.5)	4 (7.0)	4 (3.3)	0.273
Island sign		8 (4.5)	3 (5.3)	5 (4.2)	0.714
Qualitative density markers					
Heterogeneous density		29 (16.4)	17 (29.8)	12 (10.0)	0.001
Hypodensities		28 (15.8)	15 (26.3)	13 (10.8)	0.008
Swirl sign		38 (21.5)	22 (38.6)	16 (13.3)	< 0.001
Black hole sign		7 (4.0)	3 (5.3)	4 (3.3)	0.682
Blend sign		14 (7.9)	8 (14.0)	6 (5.0)	0.037
Fluid level		5 (2.8)	2 (3.5)	3 (2.5)	0.705
Quantitative density markers					
Mean ICH density (HU)		1.4 (−0.3-3.8)	4.1 (1.3–6.5)	0.5 (−0.7-2.4)	< 0.001
SD ICH density (HU)		0.3 (−0.4-1.3)	1.0 (0.3–1.7)	0.1 (−0.6-0.8)	< 0.001
CV ICH density		0.2 (−1.1-1.5)	1.1 (−0.3-1.9)	0.0 (−1.1-1.2)	< 0.001

Values are presented as n (%) or median (IQR)

*CV* coefficient of variation, *HU* Hounsfield units, *ICH* intracerebral hemorrhage, *NCCT* non-contrast computed tomography, *SD* standard deviation

^a^*p* values are from Pearson *χ*^2^, Fisher’s exact, and Mann-Whitney *U* tests

## Discussion

In the present study, heterogeneous density and hypodensities were found to be the NCCT markers that were most closely related to the dynamics of acute ICH. Heterogeneous density and hypodensities were the markers that best predicted the presence of active hemorrhage, although their performance was modest. Consistently, both heterogeneous density and hypodensities were related to a higher rate of hematoma expansion and decreased in frequency from baseline to follow-up NCCT after ICH stabilization. This decrease in frequency was higher in patients who presented active hemorrhage at baseline, supporting the value of these markers in reflecting different facets of the dynamic evolution of ICH.

NCCT markers were consistently related to ICH volume at baseline in the present study. However, ICH is a dynamic process, and hematoma expansion occurs in up to a third of ICH patients scanned within the first 6 h [23], yet the pathophysiological mechanisms of expansion remain poorly understood [6, 24]. While one model of hematoma expansion assumes ongoing bleeding for a single source, with expansion following the path of least resistance within and surrounding the hematoma, the most commonly accepted model is the so-called avalanche model [6, 8]. The avalanche model proposes that the initial vessel rupture is responsible for peripheral vessels sharing that rupture secondarily, resulting in a cascade of ruptures that maintain ongoing bleeding [8, 24, 25]. The spot sign is considered a marker of active hemorrhage [2, 23, 26, 27], specifically when detected in earlier acquisitions of CTA [3, 4]. Thus, we hypothesized that NCCT markers of hematoma expansion at baseline are related to the spot sign in phase 1 of multiphase CTA as a surrogate of active hemorrhage, and therefore, the presence of NCCT markers would decrease after ICH stabilization at follow-up NCCT, as found with heterogeneous density and hypodensities.

In the present study, mean and SD hematoma density at baseline were lower the earlier the patients were scanned. As proposed before and supporting the avalanche model, lower attenuating regions on NCCT would constitute more immature areas, and hyperattenuating regions would represent more stable areas since hematoma evolution causes extrusion of lower attenuating plasma, resulting in an even higher attenuation of the hematoma [6, 8]. Conversely, neither the presence of both heterogeneous density and hypodensities at baseline, nor the change in frequency from baseline to follow-up NCCT, was associated with a lower OIT. This reinforces the significance of these two markers at baseline in the present study, regardless of the time from symptom onset in patients scanned within the first 6 h. However, a larger multicenter study reported that their frequency may vary with OIT, even within 6 h, which may reflect differences in sample size and study design compared with the present study [28].

In contrast to other NCCT markers, the frequency of irregular shape was higher at follow-up NCCT than at baseline NCCT, despite the magnitude of the change between both NCCT scans not being related to the presence of active hemorrhage at baseline. This finding supports the hypothesis that irregular shape may reflect peripheral sites of secondary bleeding occurring at the border of the hematoma, rather than the primary source of bleeding, which is consistent with the avalanche model [6, 8, 11].

These findings have potential implications for the design of clinical trials guided by NCCT markers in patients with acute ICH [8]. Although it is thought that the different NCCT markers are actually capturing different manifestations of the same pathophysiological phenomenon [6, 14], heterogeneous density and hypodensities would be the best markers to consider, taking into account their association with different facets of the dynamic evolution of acute ICH. Furthermore, the evaluation of these two markers relies only on their visual appearance on NCCT, making them more clinically useful than quantitative density markers, black hole sign, and blend sign, which require of quantitative assessment of hematoma density [8, 9].

The strengths of this study include its prospective design, standardized neuroimaging protocol, evaluation of active hemorrhage, and standardized evaluation of NCCT markers evolution over time. The study also has some limitations, however. First, we included patients scanned within 6 h from symptom onset, what prevents the generalizability of findings to late presenters. Second, patients with uncertainty about the exact time from symptom onset were excluded, which may limit the generalizability of findings to all patients scanned within 6 h from symptom onset. Third, patients who underwent surgical hematoma evacuation or died before follow-up NCCT scan were excluded from the analysis of NCCT markers evolution, which could potentially introduce a selection bias. However, this exclusion was necessary because evaluating evolution of NCCT markers in such cases was not feasible. Fourth, we used the presence of a spot sign in phase 1 of multiphase CTA as a marker of active hemorrhage [3], but the exact pathology of the spot sign is not known, and it could represent more than just active hemorrhage. Fifth, a single rater assessed the presence of NCCT markers of hematoma expansion in the present study. However, interrater agreement was evaluated in 30 randomly selected patients by another rater and was found to be moderate-to-excellent, in line with what has been observed in previous studies [29, 30]. Sixth, markers such black hole sign, blend sign, and fluid level are infrequently or rarely found in ICH patients [16, 17, 31], which could imply that our sample size was relatively small to find associations between these markers and variables such as active hemorrhage. Finally, we did not specifically assess severe hematoma expansion, defined as an absolute increase of > 12 mL or a relative increase of > 66%, despite its recognized clinical impact [32].

## Conclusion

Heterogeneous density and hypodensities are the NCCT markers of hematoma expansion that are most closely related to the dynamics of acute ICH. Both markers are better predictors of active hemorrhage at baseline, and their presence decreases after hematoma stabilization, particularly in those patients with active hemorrhage at baseline. Conversely, the presence of irregular shape increases from baseline to follow-up NCCT, regardless of the presence of active hemorrhage at baseline, supporting the hypothesis that it may reflect peripheral sites of secondary bleeding. These findings could help in the selection of NCCT markers to guide future randomized clinical trials targeting ICH expansion.

## Supplementary Information

Below is the link to the electronic supplementary material.

Supplementary Material 1 (DOCX 214 KB)

Supplementary Material 2 (DOCX 239 KB)

## Data Availability

The data supporting this study are available from the corresponding author upon reasonable request.
