# Correction to: Temporal evolution of non-contrast CT markers of expansion relates to the dynamics of acute intracerebral hemorrhage

**DOI:** 10.1007/s00234-026-03903-2

**Published:** 2026-01-29

**Authors:** David Rodriguez-Luna, Olalla Pancorbo, João André Sousa, Renato Simonetti, Pilar Coscojuela, Marc Rodrigo-Gisbert, Federica Rizzo, Marta Olivé-Gadea, Manuel Requena, Álvaro García-Tornel, Noelia Rodriguez-Villatoro, Jesús M. Juega, Marián Muchada, Jorge Pagola, Marta Rubiera, Marc Ribo, Alejandro Tomasello, Carlos A. Molina

**Affiliations:** 1 Stroke Research Group, Vall d’Hebron Research Institute, Barcelona, Spain; 2 Stroke Unit, Department of Neurology, Vall d’Hebron University Hospital, Barcelona, Spain; 3 Department of Neuroradiology, Vall d’Hebron University Hospital, Barcelona, Spain

**Correction to: Neuroradiology**

10.1007/s00234-025-03789-6

The original version of this article unfortunately contained a mistake.

In the “Image analysis” section, the below lines contain error;

- An experienced stroke neurologist ( ) blinded to multiphase CTA scans and spot sign status prospectively evaluated the NCCT scans. Baseline and follow-up NCCT scans were evaluated in two separate reading sessions, with a minimum of 14 days between them to eliminate recall bias.
  The correct line should read;
  An experienced stroke neurologist (DRL) blinded to multiphase CTA scans and spot sign status prospectively evaluated the NCCT scans. Baseline and follow-up NCCT scans were evaluated in two separate reading sessions, with a minimum of 14 days between them to eliminate recall bias.
- An experienced neuroradiologist () blinded to follow-up NCCT scans and clinical outcomes independently inter-preted the multiphase CTA scans. The presence of spot signs was recorded in phases 1, 2, and 3 of multiphase CTA.
  The correct line should read;
  An experienced neuroradiologist (PC) blinded to follow-up NCCT scans and clinical outcomes independently inter-preted the multiphase CTA scans. The presence of spot signs was recorded in phases 1, 2, and 3 of multiphase CTA.
- To assess the interrater reliability of NCCT and mulitiphase CTA analyses, a stroke fellow () independently assessed NCCT markers of hematoma expansion, ICH volumes, and multiphase CTA spot sign status in 30 randomly selected scans.
  The correct line should read;
  To assess the interrater reliability of NCCT and mulitiphase CTA analyses, a stroke fellow (JAS) independently assessed NCCT markers of hematoma expansion, ICH volumes, and multiphase CTA spot sign status in 30 randomly selected scans.

The original article has been corrected.

The online version of the original article can be found at 10.1007/s00234-025-03789-6

