# Supplementary material for: Temporal evolution of non-contrast CT markers of expansion relates to the dynamics of acute intracerebral hemorrhage
Source: Neuroradiology. 2025 Oct 17;67(11):3195–203. doi: 10.1007/s00234-025-03789-6 (PMC12743079; doi:10.1007/s00234-025-03789-6)
Supplement: Supplementary file 2 — Supplementary Material 2 (DOCX 239 KB) [file 234_2025_3789_MOESM2_ESM.pdf]

SUPPLEMENTARY MATERIAL 2: Table S1

Article title:	Temporal Evolution of Non-Contrast CT Markers of Expansion Relates to the Dynamics of Acute Intracerebral Hemorrhage
Journal:	Neuroradiology.
Authors:	David Rodriguez-Luna, Olalla Pancorbo, João André Sousa, Renato Simonetti, Pilar Coscojuela, Marc Rodrigo-Gisbert, Federica Rizzo, Marta Olivé-Gadea, Manuel Requena, Álvaro García-Tornel, Noelia Rodriguez-Villatoro, Jesús M. Juega, Marián Muchada, Jorge Pagola, Marta Rubiera, Marc Ribo, Alejandro Tomasello, Carlos A. Molina.
Correspondence:	David Rodriguez-Luna. Stroke Research Group, Vall d'Hebron Research Institute (VHIR). Ps. Vall d'Hebron, 119, 08035, Barcelona, Spain. Phone: +34 934893000 (6363). Email: david.rodriguez@vhir.org

Table S1. Frequency of shape and qualitative density markers and median values of quantitative density markers at baseline according to the presence of the spot sign in phases 1, 2, and 3 of multiphase CTA

	Phase 1 (n=232)			Phase 2 (n=215)			Phase 3 (n=215)		
	Yes (n=84)	No (n=148)	*p* Value[a]	Yes (n=95)	No (n=120)	*p* Value[a]	Yes (n=100)	No (n=115)	*p* Value[a]
**Shape markers**									
**Irregular shape**	69 (82.1)	89 (60.1)	<0.001	75 (78.9)	72 (60.0)	0.003	79 (79.0)	68 (59.1)	0.002
**Satellite sign**	28 (33.3)	25 (16.9)	0.004	30 (31.6)	20 (16.7)	0.010	30 (30.0)	20 (17.4)	0.029
**Island sign**	55 (65.5)	60 (40.5)	<0.001	59 (62.1)	48 (40.0)	0.001	61 (61.0)	46 (40.0)	0.002
**Qualitative density markers**									
**Heterogeneous density**	37 (44.0)	31 (20.9)	<0.001	34 (35.8)	30 (25.0)	0.086	36 (36.0)	28 (24.3)	0.062
**Hypodensities**	53 (63.1)	56 (37.8)	<0.001	57 (60.0)	45 (37.5)	0.001	60 (60.0)	42 (36.5)	0.001
**Swirl sign**	52 (61.9)	60 (40.5)	0.002	55 (57.9)	50 (41.7)	0.018	58 (58.0)	47 (40.9)	0.012
**Black hole sign**	7 (8.3)	4 (2.7)	0.103	6 (6.3)	5 (4.2)	0.478	5 (5.0)	6 (5.2)	0.942
**Blend sign**	12 (14.3)	13 (8.8)	0.194	10 (10.5)	12 (10.0)	0.899	12 (12.0)	10 (8.7)	0.425
**Fluid level**	2 (2.4)	4 (2.7)	0.999	2 (2.1)	2 (1.7)	0.999	2 (2.0)	2 (1.7)	0.999
**Quantitative density markers**									
**Mean ICH density (HU)**	63.0 (60.1-65.2)	63.8 (60.2-66.7)	0.138	62.9 (60.3-65.3)	63.8 (60.2-66.5)	0.199	62.8 (60.3-65.4)	63.9 (60.2-66.5)	0.199
**SD ICH density (HU)**	8.8 (8.0-9.5)	9.3 (8.6-10.1)	<0.001	8.9 (8.1-9.7)	9.2 (8.5-10.0)	0.024	8.9 (8.1-9.7)	9.2 (8.5-10.0)	0.043
**CV ICH density**	14.2 (13.1-15.0)	14.6 (13.4-15.9)	0.010	14.2 (13.1-15.3)	14.6 (13.4-15.8)	0.122	14.3 (13.1-15.3)	14.5 (13.3-15.8)	0.232

Values are presented as n (%) or median (IQR).

CV, coefficient of variation; *HU*, Hounsfield units; *ICH*, intracerebral hemorrhage; *NCCT*, non-contrast computed tomography; *SD*, standard deviation.

[a]*p* values are from Pearson χ^2, Fisher's exact, and Mann-Whitney *U* tests.
